# Supplementary material for: CryoEM structures of Kv1.2 potassium channels, conducting and non-conducting
Source: eLife. 2025 Feb 13;12:RP89459. doi: 10.7554/eLife.89459 (PMC11825129; doi:10.7554/eLife.89459)
Supplement: Supplementary file 1. [file elife-89459-supp1.docx]

|  | Kv1.2-open | Kv1.2-W366F | Kv1.2-DTx | Kv1.2-Na^+^ | Kv1.2-W366F-Na+ |
| --- | --- | --- | --- | --- | --- |
| **Data collection and processing** | | | | | |
| Microscope | FEI Titan Krios | | | | |
| Detector | K3 | | | | |
| Voltage (kV) | 300 | | | | |
| Electron exposure (e-/Å^2) | 50 | | | | |
| Magnification | 81000 | | | | |
| Defocus range (um) | -1.0 to -2.0 | | | | |
| Pixel Size (Å/pixel) | 1.068 | | | | |
| Symmetry imposed | C4 | C4 | C1 | C4 | C4 |
| Micrographs (no.) | 6335 | 8310 | 4507 | 2573 | 9057 |
| Map resolution (Å) | 3.2 | 2.5 | 3.2 | 2.8 | 7.8 |
| Final particle images (no.) | 228519 | 266495 | 312202 | 433211 | 68682 |
| FSC threshold | 0.143 | | | | |
|  |  |  |  |  |  |
| **Refinement** | | | | | |
| PDB codes | 6VC6 | 6VCH | 6VC3 | 6VC4 |  |
| EMDB codes | 43134 | 43136 | 43131 | 43133 |  |
| Map sharpening B factor (Å^2) | -185.2 | -50 | -116.8 | -157.8 |  |
| Model Composition | | | | |  |
| Chains | 4 | 5 | 6 | 4 |  |
| Atoms | 7740 | 7614 | 8219 | 7740 |  |
| Residues | 1028 | 1012 | 1087 | 1028 |  |
| Water | 0 | 0 | 0 | 0 |  |
| Ligands | 0 | K: 2 | K: 2 | 0 |  |
| Bonds (RMSD) | | | | |  |
| Length (Å) | 0.003 (0) | 0.004 (0) | 0.002 (0) | 0.003 (0) |  |
| Angles (°) | 0.562 (0) | 0.577 (0) | 0.484 (0) | 0.621 (0) |  |
| Validation | | | | |  |
| Molprobity score | 1.52 | 1.37 | 1.43 | 1.55 |  |
| Clashscore | 7.58 | 5.83 | 7.03 | 8.1 |  |
| Poor rotamers (%) | 0.26 | 0.65 | 0.24 | 0.56 |  |
| Ramachandran plot | | | | |  |
| Favoured (%) | 97.51 | 97.77 | 97.83 | 97.91 |  |
| Allowed (%) | 2.49 | 2.23 | 2.17 | 2.09 |  |
| Outliers (%) | 0.00 | 0.00 | 0.00 | 0.00 |  |

Supplementary File 1. CryoEM data collection, refinement and validation statistics.
